# Metallurgical Coke Combustion with Different Reactivity under Nonisothermal Conditions: A Kinetic Study

**DOI:** 10.3390/ma15030987

**Published:** 2022-01-27

**Authors:** Yuelin Qin, Qingfeng Ling, Wenchao He, Jinglan Hu, Xin Li

**Affiliations:** 1 School of Metallurgy and Materials Engineering, Chongqing University of Science and Technology, Chongqing 401331, China; lingqingfeng9762@163.com (Q.L.); hewenchao1217@163.com (W.H.); hujinglan_cqust@163.com (J.H.); lixin2020202034@163.com (X.L.); 2 Value-Added Process and Clean Extraction of Complex Metal Mineral Resources, Chongqing Municipal Key Laboratory of Institutions of Higher Education, Chongqing 401331, China

**Keywords:** metallurgical coke, combustion characteristics, Coats–Redfern, kinetics, reactivity

## Abstract

The combustion characteristics and kinetics of high- and low-reactivity metallurgical cokes in an air atmosphere were studied by thermogravimetric instrument. The Coats–Redfern, FWO, and Vyazovkin integral methods were used to analyze the kinetics of the cokes, and the kinetic parameters of high- and low-reactivity metallurgical cokes were compared. The results show that the heating rate affected the comprehensive combustion index and combustion reaction temperature range of the cokes. The ignition temperature, burnout temperature, combustion characteristics, and maximum weight-loss rate of low-reactivity coke (L-Coke) were better than high-reactivity coke (H-Coke). Low-reactivity coke had better thermal stability and combustion characteristics. At the same time, it was calculated via three kinetic analysis methods that the combustion activation energy gradually decreased with the progress of the reaction. The coke combustion activation energy calculated by the Coats–Redfern method was larger than the coke combustion activation energy calculated by the FWO and Vyazovkin methods, but the laws were consistent. The activation energy of L-Coke was about 4~8 kJ/mol more than that of H-Coke.

## 1. Introduction

The iron and steel industry is energy intensive and is the second largest user of energy in the world industrial sector. Its carbon dioxide (CO_2_) emissions account for approximately 33.8% of total industrial CO_2_ emissions and 7% of total global CO_2_ emissions [1,2]. As part of the iron and steel industry, the ironmaking system produces about 70% of the total CO_2_ emissions of steel [3]. In order to reduce CO_2_ emissions from the ironmaking system, researchers have successively proposed a series of low-carbon ironmaking solutions based on hydrogen reduction, such as blast-furnace injection of hydrogen-bearing materials, coal-dust fuel and natural gas, the “Ultra-Low CO_2_ Steelmaking” (ULCOS) emissions project, and “CO_2_ ultimate reduction in steel-making process by innovative technology for cool Earth 50” (COURSE 50) [4,5,6,7].

Metallurgical coke is an indispensable raw material for blast-furnace smelting. Its role in the blast furnace is to provide heat for chemical reactions, act as a reducing agent for iron ore, increase the carbon content in molten iron, maintain the stability of the blast-furnace column, and support the flow of gas up and down. [8,9,10]. In the lower part of the blast furnace, after the iron ore is reduced and dripped, coke becomes the only lumpy material. It is not only affected by factors such as temperature, CO_2_, slag, molten iron, and gas flow, but also needs to ensure its stability, support the upper charge of the blast furnace, ensure that the blast-furnace column does not collapse, and produce smoothly [11,12]. Therefore, the quality of metallurgical coke is closely related to the technical and economic indicators of blast furnaces. Mansheng et al. found that from 1100 °C to 1500 °C, the coke weight-loss ratio increased by 10-fold, and the drum strength decreased by 80%; in a CO_2_ atmosphere, when the temperature increased from 1100 °C to 1300 °C, the coke reactivity increased by 50%, and the coke strength decreased after the reaction [13]. Zhongsuo used different methods to analyze the kinetics of carbon dioxide gasification of metallurgical coke, and proposed new kinetic equations and predicted kinetic curves [14]. Qi et al. studied the effect of the Stefan flow on coke dissolution with metallurgical cokes of low, medium, and high reactivity [15]. It is generally believed that the reactive CRI of coke is inversely proportional to the post-reaction strength CSR [16]. Under high temperature and CO_2_ atmosphere detection, high-reactivity coke often shows a decrease in strength and loose structure, which is inconsistent with the skeleton support and good permeability of the blast furnace [17]. Therefore, low-reactivity coke is often used in the blast-furnace smelting process to ensure the blast-furnace framework function of the coke, but some steel companies use high-reactivity coke for hydrogen-rich smelting in blast furnaces and blast furnaces can still operate stably [18,19], so the practical application of high-reactivity coke is thought-provoking. Based on the above analysis, this paper uses a thermogravimetric analyzer to study the combustion behavior and kinetics of high- and low-reactivity coke.

## 2. Experimental

### 2.1. Materials

The metallurgical coke used in the experiment was obtained from Chongqing Iron and Steel Company in Chongqing and Xinjiang Bayi Iron and Steel Company in Xinjiang, China. Tested by national standards (GB/T 4000-2017), the CRI of low-reactivity coke is 25.2%, and the CRI of high-reactivity coke is 53.4%. Low-reactivity coke and high-reactivity coke are abbreviated as L-Coke and H-Coke, respectively. The proximate analysis (GB/T 2001-2013) and ultimate analysis (GB/T213-2003) of metallurgical coke within 20–40 mm is illustrated in Table 1.

### 2.2. Experimental Device

The thermogravimetric analyzer (HTG-2) produced by Beijing Hengjiu Scientific Instrument Factory (Beijing, China) was used. The thermogravimetric (TG) curve was selected, and the curve was differentiated to obtain the corresponding derivative thermogravimetry (DTG) curve for analysis. The test temperature range of the thermogravimetric analyzer was from room temperature to 1250 °C, the temperature accuracy was ±0.1 °C, the heating rate was 0.1~80 °C/min, the measurement range of the sample was 0~300 mg, and the mass accuracy was 0.1 μg. The Al_2_O_3_ crucible was selected in combination with the nature of the sample itself to ensure that the sample and the crucible did not react at high temperature, and to avoid the inaccuracy of the experimental data and the safety of the instrument. In this experiment, the sample was raised to 1100 °C from room temperature with heating rates of 5 °C/min, 10 °C/min, 15 °C/min, and 20 °C/min in the atmosphere.

## 3. Results and Discussion

### 3.1. Combustion Characteristics

Combustion characteristics are usually used to judge the burning speed and thermal stability of the sample. Through the calculation and analysis of TG and DTG curves, the combustion characteristic indexes of the coke can be obtained, which are mainly ignition temperature (T_i_), burnout temperature (T_b_), maximum weight-loss rate (v_max_), and comprehensive combustion characteristic index (S) [20].

**Figure 1:**
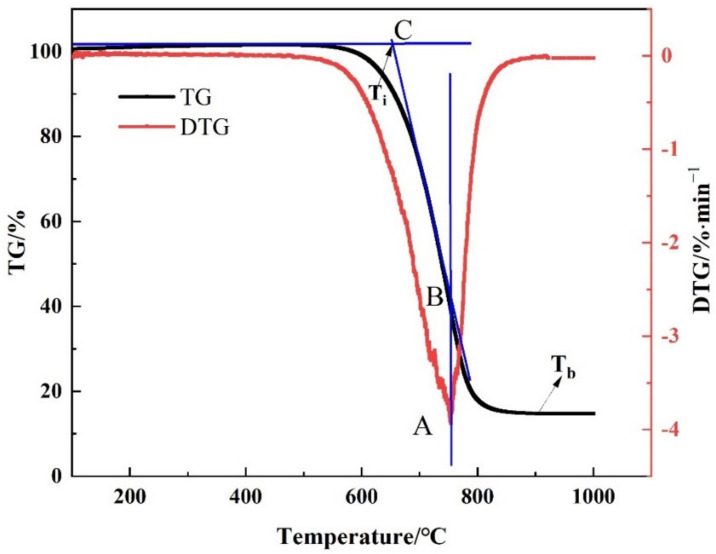
Schematic diagram for the combustion characteristic temperature of coke (T_i_ is the ignition temperature, T_b_ is the burnout temperature, A is the peak point of the DTG curve, B is the point with the fastest weight loss rate, C is the intersection of the tangent line passing through point B and the horizontal line where weightlessness begins).

Firstly, T_i_ refers to the temperature at which the coke begins to burn. As shown in Figure 1, it can be calculated by the TG-DTG curve—that is, a vertical line is drawn at the peak point A of the DTG curve, intersecting with the TG curve at point B, and the tangent of the TG curve through point B. The tangent starts with the weight loss. The temperature corresponding to the intersection point C of the parallel lines is defined as the T_i_ [21]. Secondly, T_b_ is the temperature at which the coke combustion ends. The temperature at the point where the tail end of the DTG curve is approximately parallel to the X axis is defined as the T_b_. Finally, v_max_ is an important parameter of the characteristics of the reaction coke, corresponding to the point where the reaction rate is the fastest in the weight-loss process, and the v_max_ is A at the lowest peak point of the DTG curve. The calculation formula of S is as shown in Equation (1) [21]:

(1) $$S=\frac{v_{\max}\;\times \;v_{\mathrm{mean}}}{T_{i}^{2}\;\times \;T_{b}}$$

where T_i_ is the ignition temperature of the coke, expressed as °C; T_b_ is the burnout temperature of the coke, expressed as °C; v_max_ is the maximum combustion rate of the coke, expressed as %/min; and v_mean_ is the average combustion rate of the coke, expressed as %/min.

S is used to describe the comprehensive combustion performance of coke. The larger the comprehensive combustion characteristic index is, the better the combustion characteristic of the coke is.

**Figure 2:**
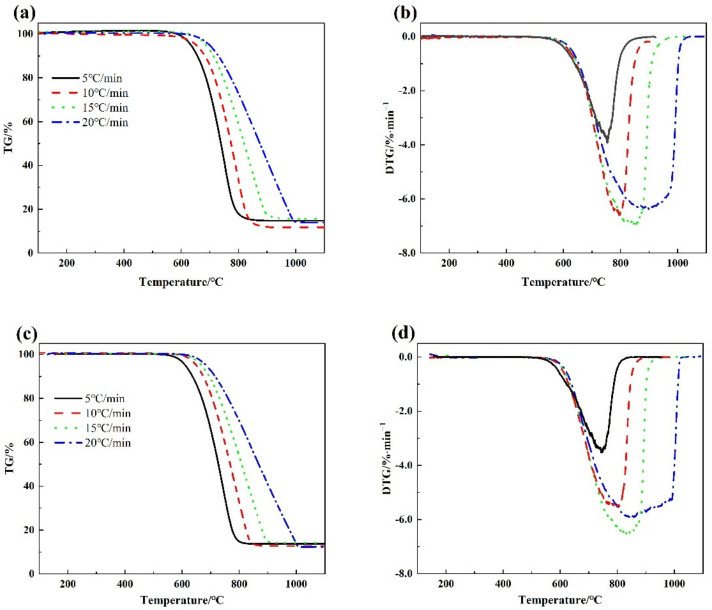
TG-DTG curves of metallurgical coke combustion with different reactivity (**a**) is the TG curve of the L-Coke, (**b**) is the DTG curve of the L-Coke, (**c**) is the TG curve of the H-Coke (**d**) is the DTG curve of the H-Coke.

Figure 2 shows that the combustion temperature range of metallurgical coke is between 600 °C and 1000 °C. It can be seen from the TG curve that with the increase in the heating rate, the slope of the TG curve of the coke became smaller, the combustion temperature range increased, and the burnout temperature increased. It can be seen from the DTG curve that when both cokes were at 15 °C/min, the instantaneous change was the largest, indicating that the coke burned with the fastest weight loss at this combustion rate.

**Figure 3:**
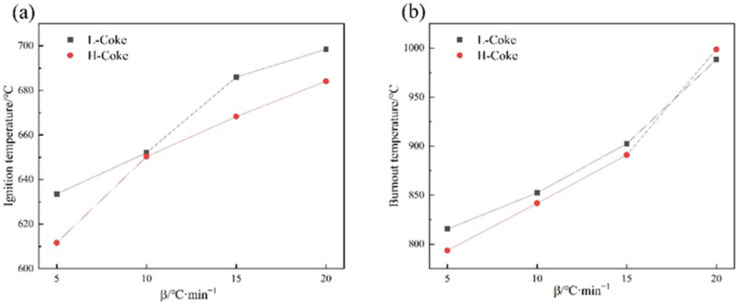

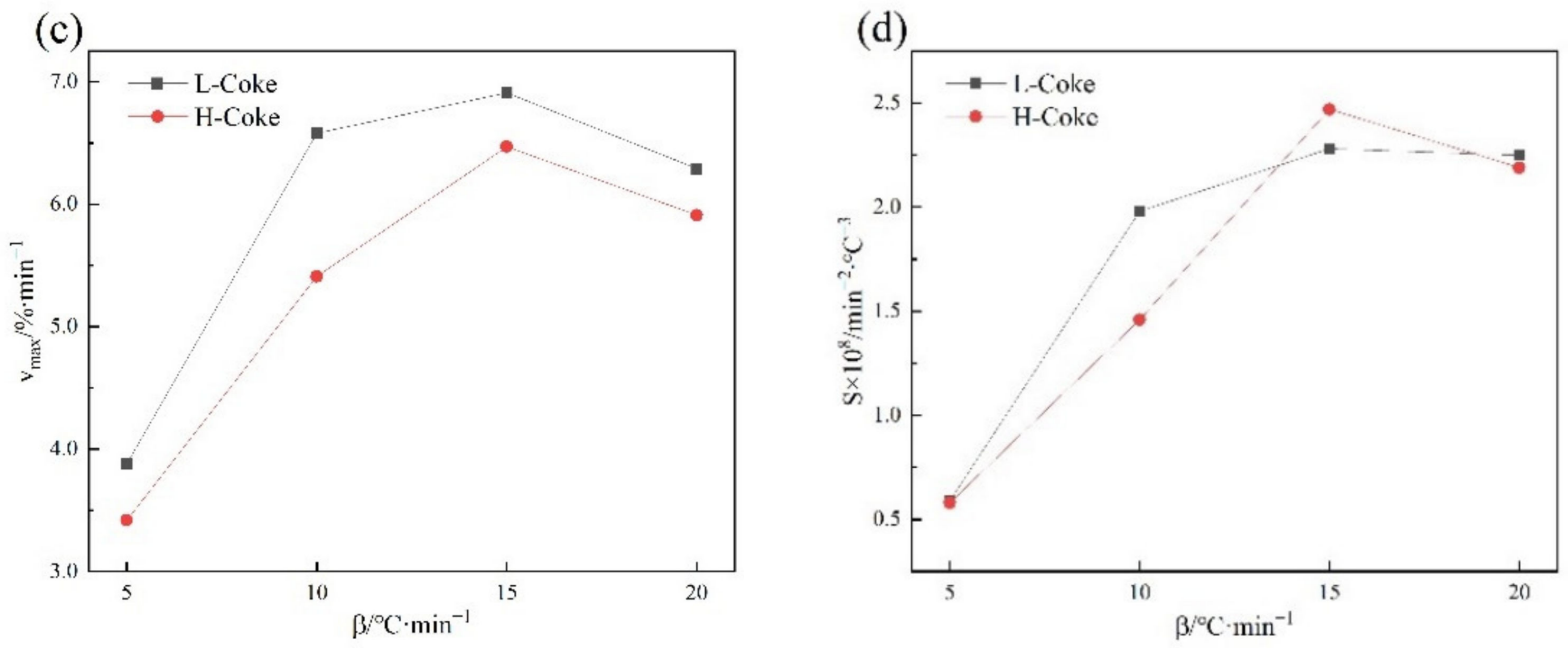
Combustion characteristic parameters of metallurgical coke at different heating rates (**a**) is the relationship between ignition temperature (T_i_) and heating rate, (**b**) is the relationship between burnout temperature (T_b_) and heating rate, (**c**) is the relationship between maximum weight-loss rate (v_max_) and heating rate, (**d**) is the relationship between comprehensive combustion characteristic index (S) and heating rate.

Figure 3 shows the combustion characteristic parameters of metallurgical coke at different heating rates. With the increase in the heating rate, all combustion characteristic parameters increased, among which the L-Coke was higher than the H-Coke.

Table 2 shows that with the increase in the heating rate, the T_i_ of L-Coke increased by 64.85 °C, the T_b_ increased by 172.73 °C, and the S increased by 1.69, and the Ti of H-Coke increased by 72.47 °C, the T_b_ increased by 205.13 °C, and the S increased by 1.61, indicating that the heating rate had a greater impact on L-Coke and H-Coke had better thermal stability.

In addition, when the heating rate β = 15 °C/min, the maximum reaction rate of the two cokes reached the peak, and the S also reached the peak, indicating that the combustion characteristics of the coke were the best at this heating rate. Finally, the T_i_ and T_b_ of coke all shifted to high temperature with the increase in heating rate, and the maximum weight loss rate decreased slightly. The reason is that the heat provided by the outside could not be transferred from the surface of the coke to the inside of the coke in time, resulting in the occurrence of thermal hysteresis.

### 3.2. Kinetic Analysis

By comparing the combustion TG curves of the two cokes, it was found that there were differences in thermal stability. The kinetic parameters were obtained by thermal analysis kinetics [22]. Usually the mode function, activation energy (E_a_), and pre-exponential factor (A) are defined as kinetic parameters. Among them, the E_a_ characterizes the difficulty of the reaction and reflects the minimum energy required for the reactant molecules to reach the activated state during the chemical reaction; the greater the activation energy, the more difficult it is to proceed, and vice versa. The pre-referential factor indicates the number of molecules that effectively collide, that is, the extent of the chemical reaction per unit time.

**Figure 4:**
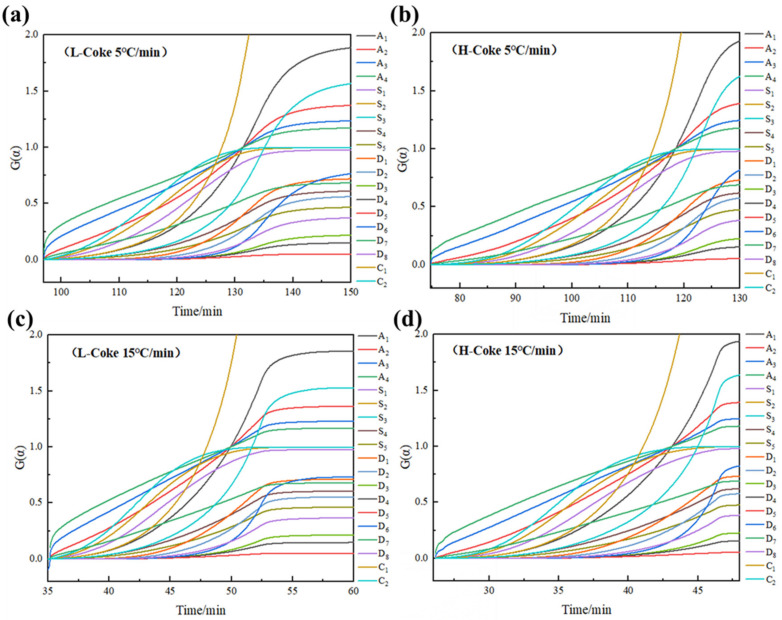
Kinetic models of coke combustion reactions at 5 °C/min and 15 °C/min (**a**) is the L-Coke combustion at 5 °C/min, (**b**) is the H-Coke combustion at 5 °C/min, (**c**) is the L-Coke combustion at 15 °C/min, (**d**) is the H-Coke combustion at 15 °C/min.

The equal conversion method and integral method are used to obtain the activation energy of coke combustion. The differential and integral functions of these theoretical models are detailed in Table 3. In this study, 19 kinetic models were investigated, as shown in Table 3. The G(α) versus t-plots were first established using the mechanism functions, including the nucleation and growth mechanism, chemical reaction, and mass diffusions, as presented in Figure 4 [23]. g(α) is the reaction mechanism function, and G(α) is the integral form of g(α).

Generally, non-isothermal reactions can be regarded as infinitely many isothermal reactions in the integral definition. The kinetic equation of the isothermal method is G(α) = kt, where k is the rate constant. The two types of coke were fitted to 19 mode functions at 5 °C/min and 15 °C/min, as shown in Figure 4. A straight line was obtained from the plot of G(α) and t. The G(α), which makes the linearity of the straight line the best, was determined to be an appropriate mechanism function.

The results demonstrate that an A_1_ model (Avrami–Erofeev, m = 1) interpreted the kinetic mechanism most reasonably, as described by G(α)= −ln(1 − α). In fact, Avrami–Erofeev models were generally used to interpret the gas–solid reactions when the porosity of the solids varied during the reactions, the rate-controlling step of which is the nucleation step [26,27]. However, the approximate isothermal method has certain limitations, so it was necessary to use the non-isothermal method of single scan rate and multiple scan rate to check again.

#### 3.2.1. Coats–Redfern Method

The Coats–Redfern method is a non-isothermal method for thermal kinetics analysis of experimentally measured TG curve data at a fixed heating rate, belonging to a single scan rate method [28]. Its kinetic equation is shown in Equation (2):

(2) $$\ln\left[\frac{G(\alpha )}{T^{2}}\right]=\ln\left[\frac{\mathrm{AR}}{\beta E_{a}}(1-\frac{2\mathrm{RT}}{E_{a}})\right]-\frac{E_{a}}{\mathrm{RT}}$$

where α is the conversion rate, defined as $\alpha =\frac{m_{0}-m_{t}}{m_{0}-m_{1}}$, expressed as %; *m*_0_ is the initial mass of the sample, expressed as mg;* m_t_* is the mass of the sample at a certain moment in the weight-loss process, expressed as mg; *m*_1_ is the mass of the sample after reaction, expressed as mg; G(α) is the mechanism function; A is the pre-exponential factor, expressed as min^−1^; E_a_ is the activation energy, expressed as kJ/mol; R is the gas constant, expressed as 8.314 × 10^3^ kJ/(mol·K); and β is the heating rate, expressed as °C/min.

In the combustion process of the coke sample, the result of (2RT/E_a_) was much less than 1 and the result of (1 − 2RT/E_a_) was about equal to 1. Equation (2) can be represented as

(3) $$\ln\left[\frac{G(\alpha )}{T^{2}}\right]=\ln\left[\frac{\mathrm{AR}}{\beta E_{a}}\right]-\frac{E_{a}}{\mathrm{RT}}$$

A straight line can be obtained from the plot of ln[G(α)/T^2^] and 1/T. Here, the A and E_a_ values can be obtained from the slope and intercept of the line.

In order to further clarify the effect of the mechanism function on the kinetics of coke combustion, the idea of segmentation was adopted, and the interval was divided into two sections based on the conversion rate. The kinetic curve analysis of ln[G(α)/T^2^] vs. 1/T of a mechanism function was used to determine the optimal mechanism function with the value of the correlation coefficient R^2^, as shown in Table 4. After fitting, in the first half of the temperature range, chemical reaction (*n* = 2) was the best mechanism function; in the second half of the temperature range, Avrami–Erofeev (m = 1) was the best mechanism function. Their integral forms are G(α) = (1 − α)^−1^ – 1 and G(α) = −ln(1 − α), respectively. The kinetic equations expressed in Coats–Redfern are shown in Equations (4) and (5).

When

(4) $$\ln\left[\frac{{(1\;-\;\alpha )}^{-1}-\;1}{T^{2}}\right]=\ln\left[\frac{\mathrm{AR}}{\beta E_{a}}\right]-\frac{E_{a}}{\mathrm{RT}}$$

When

(5) $$\ln\left[\frac{-\;\ln(1\;-\;\alpha )}{T^{2}}\right]=\ln\left[\frac{\mathrm{AR}}{\beta E_{a}}\right]-\frac{E_{a}}{\mathrm{RT}}$$

Table 4 shows the kinetic parameters of the coke calculated by the Coats–Redfern method. The research shows that the apparent activation energy in the low-temperature region was equal to the actual activation energy in the coke combustion process, the apparent activation energy in the medium-temperature region was smaller than the actual activation energy, and the apparent activation energy in the high-temperature region was equal to zero. When calculating the coke kinetic parameters, the combustion process was divided into two regions: the low-temperature section and the high-temperature section. The kinetic correlation coefficient R^2^ of the double temperature range was above 0.99, indicating that the regression effects are highly significant and credible.

When the heating rate was 5 °C/min, the activation energy of L-Coke was 266.1568 kJ/mol and the activation energy of H-Coke was 265.7327 kJ/mol. When the heating rate was 20 °C/min, the activation energy of L-Coke was 207.9613 kJ/mol and the activation energy of H-Coke was 194.6635 kJ/mol. When the heating rate increased, the activation energy decreased, and the pre-exponential factor A also decreased. The average activation energy of L-Coke and H-Coke were 249.2651 kJ/mol and 244.9541 kJ/mol, respectively. At different heating rates, the activation energy of L-Coke was also higher than that of H-Coke.

#### 3.2.2. Equal Conversion Rate Method

The multiple scanning rate method is called the equal conversion rate method, which refers to the kinetic analysis method using the data at the same conversion rate α on the TG curve under different heating rates. It can obtain a reliable E_a_ without involving kinetic mode functions, can verify the results of the Coats–Redfern method, and can verify the consistency of the reaction mechanism throughout the process. Therefore, the Flynn–Wall–Ozawa (FWO) and Vyazovkin integral methods [29,30] were used for calculation, and their kinetic equations are shown in Equations (6) and (7), respectively.

(6) $$\ln\beta =\ln\left[\frac{{\mathrm{AE}}_{a}}{\mathrm{RG}(\alpha )}\right]-5.331-1.052\frac{E_{a}}{\mathrm{RT}}$$

(7) $$\ln\frac{\beta }{T^{2}}=\ln\frac{\mathrm{AR}}{{G(\alpha )E}_{a}}-\frac{E_{a}}{\mathrm{RT}}$$

Since the same α is selected under different heating rates, G(α) is a constant value. Therefore, ln[AE_a_/RG(α)] and ln[AR/G(α) E_a_] are constant values. Lnβ and ln(β/T^2^) have a linear relationship with 1/T, they are fitted to a straight line, and the slopes are −1.052E_a_/R and -E_a_/R. Therefore, the E_a_ is obtained from the slope.

**Figure 5:**
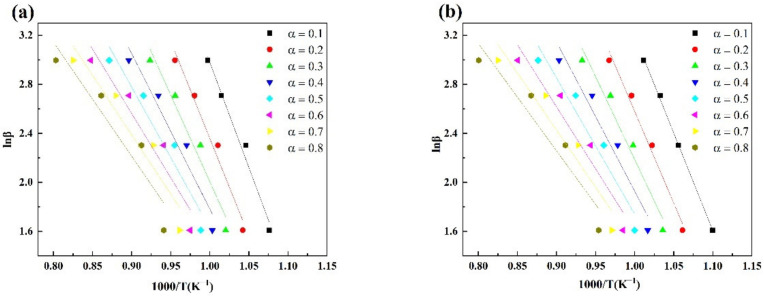
Fitting curves of lnβ vs. 1/T of coke based on the FWO method (**a**) L-Coke, (**b**) H-Coke.

**Figure 6:**
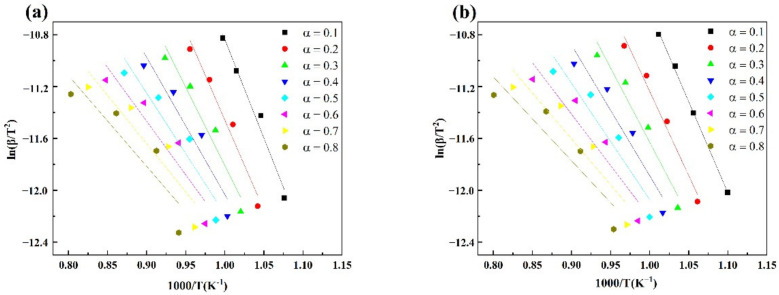
Fitting curves of ln(β/T^2^) vs. 1/T of coke based on the Vyazovkin method (**a**) L-Coke, (**b**) H-Coke.

Table 5 shows the coke combustion kinetic parameters calculated with the FWO and Vyazovkin methods. R^2^ is the linear correlation coefficient. Figure 5 and Figure 6 are the linear fitting results at different conversion rates obtained by the Vyazovkin and FWO kinetic analysis methods. It can be seen from Table 5 that the activation energy calculated by the two methods decreased with the increase in the conversion rate. When the conversion rate was 0.1–0.8, the activation energy of the H-Coke was always lower than that of the L-Coke. This shows that it was more difficult to get the L-Coke to start burning, which also confirms that the L-Coke had better combustion characteristics. The low-temperature section corresponds to the medium- and low-temperature regions of coke combustion and includes a part with a conversion rate of 0.1 to 0.4. Therefore, the average value of the activation energy with a conversion rate of 0.1 to 0.4 was taken as the activation energy of coke. Calculated by the FWO method, the average activation energy of the L-Coke was 95.5590 kJ/mol and the average activation energy of the H-Coke was 91.5119 kJ/mol. Calculated by the Vyazovkin method, the average activation energy of the L-Coke was 87.8103 kJ/mol and the average activation energy of the H-Coke was 83.4133 kJ/mol. Because the combustion reaction rate of coke is related to the diffusion reaction rate and the oxidation reaction rate, the combustion interface between coke and oxygen reacts violently and the reactants at the combustion interface rapidly diffuse out. The coke core shrinks and the specific surface area decreases, resulting in a decrease in activation energy. In addition, the activation energy calculated by the FWO method was larger than the activation energy calculated by the Vyazovkin method, because the two cleverly avoided the influence of the mechanism function on the activation energy, and the temperature integral approximation used was different, resulting in the deviation in the obtained values. However, the activation energy law was the same. In summary, highly reactive coke has lower activation energy than low-reactive coke and is easier to burn, which is also consistent with the results obtained by the Coats–Redfern method.

## 4. Conclusions

(1). With the increase in the heating rate, the ignition temperature and burnout temperature of the two cokes increased, the combustion time was shortened, the comprehensive combustion characteristic index increased, and the combustion characteristics were improved. Low-reactivity coke had better thermal stability and combustion characteristics.
(2). With the increase in the heating rate, the activation energy of coke combustion obtained by the Coats–Redfern method gradually decreased, and the activation energy of L-Coke was about 4 kJ/mol more than that of H-Coke.
(3). The activation energy calculated by the FWO method was higher than that calculated by the Vyazovkin method, but the laws obtained by the two methods were the same. The activation energy of L-Coke was about 8 kJ/mol higher than that of H-Coke.
(4). The coke combustion kinetic parameters provide the basic data parameters for the numerical simulation of blast furnace pre-tuyere combustion and provide the basis for the application of high-reactivity coke in blast furnaces.

## Figures and Tables

**Table 1:** Proximate and ultimate analysis results of metallurgical coke, wt%.

Sample	Proximate Analysis				Ultimate Analysis				
	M_ad_	V_ad_	A_d_	FC_d_	C	H	O	N	S
L-Coke	0.92	1.32	12.80	84.96	86.32	0.45	0.88	1.87	0.48
H-Coke	0.68	1.18	11.66	86.48	87.68	0.28	0.62	1.06	0.36

Note: ad—air dry basis; M—moisture; V—volatile matter; A—ash; FC—fixed carbon.

**Table 2:** Combustion characteristic parameters of metallurgical coke at different heating rates.

Sample	β (°C/min)	T_i_ (°C)	T_b_ (°C)	v_max_ (%/min)	v_mesn_ (%/min)	S × 10^8^ (min^−2^/°C^3^)
L-Coke	5	633.56	815.52	3.88	0.50	0.59
	10	651.99	852.28	6.58	1.09	1.98
	15	686.00	902.55	6.91	1.38	2.28
	20	698.41	988.25	6.29	1.75	2.25
H-Coke	5	611.64	793.55	3.42	0.50	0.58
	10	650.33	841.72	5.41	0.96	1.46
	15	668.27	891.03	6.47	1.52	2.47
	20	684.11	998.68	5.91	1.73	2.19

**Table 3:** Common mechanism functions in gas–solid reactions [23,24,25] (Adapted with permission from ref. [23,24,25], 2012 and 2013 Peng, L and 2015 Sun, Y).

No.	Reaction Mechanism	g(α)	G(α)
A_1_	Avrami–Erofeev, (m = 1)	1 − α	− ln(1 − α)
A_2_	Avrami–Erofeev, (m = 2)	2(1 − α) [− ln(1 − α)]^1/2^	[− ln(1− α)]^1/2^
A_3_	Avrami–Erofeev, (m = 3)	3(1 − α) [− ln(1 − α)]^2/3^	[− ln(1− α)]^1/3^
A_4_	Avrami–Erofeev, (m = 4)	4(1 − α) [− ln(1 − α)]^3/4^	[− ln(1− α)]^1/4^
S_1_	Shrinking core, (m = 1/2)	1/2 (1 −α) ^−1^	1−(1 − α)^2^
S_2_	Shrinking core, (m = 1/3)	1/3 (1 −α)^−2^	1−(1 − α)^3^
S_3_	Shrinking core, (m = 1/4)	1/4 (1 − α)^−3^	1−(1 − α)^4^
S_4_	Shrinking core, (m = 2)	2 (1 − α)^1/2^	1 − (1 − α)^1/2^
S_5_	Shrinking core, (m = 3)	3 (1 − α)^2/3^	1 − (1 − α)^1/3^
D_1_	One-dimensional	1/2α^−1^	α^2^
D_2_	Two-dimensional	[−ln(1 − α)]^−1^	α + (1 − α) ln(1 − α)
D_3_	Three-dimensional	3/2(1 − α)^2/3^[1 − (1 − α)^1/3^]^−1^	[1 − (1 − α)^1/3^]^2^
D_4_	Three-dimensional	3/2[(1 − α)^−1/3^ − 1]^−1^	1 − 2/3α − (1 − α)^2/3^
D_5_	3-D (anti-Jander)	3/2(1 + α)^2/3^[(1 + α)^1/3^ − 1]^−1^	[(1 + α)^1/3^ − 1]^2^
D_6_	3-D (ZLT)	3/2(1 − α)^4/3^[(1 − α)^1/3^ − 1]^−1^	[(1 − α)^−1/3^ − 1]^2^
D_7_	3-D (Jander)	6(1 − α)^2/3^[1 − (1 − α)^1/3^]^1/2^	[1−(1 − α)^1/3^]^1/2^
D_8_	2-D (Jander)	(1 − α)^1/2^[1 − (1 − α)^1/2^]^2^	[1 − (1 − α)^1/2^]^2^
C_1_	Chemical reaction, (*n* = 2)	(1 − α)^2^	(1 − α)^−1^ − 1
C_2_	Chemical reaction, (*n* = 3/2)	(1 − α)^2/3^	(1 − α)^−1/2^ − 1

**Table 4:** Kinetic parameters of the coke calculated by the Coats–Redfern method.

Sample	β/(°C/min)	T/°C	E_a_/(kJ/mol)	A/(min^−1^)	R^2^
L-Coke	5	633.56~707.42	266.1568	7.69 × 10^9^	0.9953
		707.43~815.52	148.4800	2.79 × 10^6^	0.9972
	10	651.99~739.28	264.1843	3.83 × 10^9^	0.9974
		739.29~852.28	149.4101	3.22 × 10^6^	0.9999
	15	686.00~773.18	258.7578	2.33 × 10^8^	0.9904
		773.19~902.55	113.0397	2.97 × 10^5^	0.9998
	20	698.41~809.36	207.9613	4.02 × 10^6^	0.9970
		809.37~988.25	72.4484	1.90 × 10^3^	0.9994
H-Coke	5	611.64~692.51	265.7327	5.56 × 10^9^	0.9982
		692.52~793.55	137.7137	1.97 × 10^6^	0.9998
	10	650.33~728.79	262.9306	2.90 × 10^9^	0.9993
		728.80~841.72	125.0834	1.80 × 10^6^	0.9998
	15	668.27~758.68	256.4894	2.18 × 10^8^	0.9965
		758.69~891.03	103.0143	1.04 × 10^5^	0.9998
	20	684.11~799.82	194.6635	4.92 × 10^6^	0.9973
		799.83~998.68	71.4838	1.71 × 10^3^	0.9997

**Table 5:** Kinetic parameters of coke based on the FWO and Vyazovkin methods.

Sample	α	FWO		Vyazovkin	
		E/(kJ·mol^−1^)	R^2^	E/kJ·mol^−1^	R^2^
L-Coke	0.1	123.6115	0.9959	119.8789	0.9946
	0.2	99.8798	0.9977	92.7443	0.9966
	0.3	84.8729	0.9906	75.6199	0.9850
	0.4	73.8716	0.9875	62.9981	0.9781
	0.5	65.3441	0.9845	53.1409	0.9700
	0.6	58.5082	0.9839	45.1669	0.9652
	0.7	53.2430	0.9821	38.9299	0.9565
	0.8	49.6779	0.9821	34.5463	0.9516
Average (0.1 ~ 0.4)		95.5590		87.8103	
H-Coke	0.1	111.2866	0.9948	106.0545	0.9931
	0.2	99.5973	0.9855	92.6350	0.9791
	0.3	83.0341	0.9739	73.7578	0.9592
	0.4	72.1295	0.9703	61.2059	0.9492
	0.5	64.2675	0.9676	52.0476	0.9390
	0.6	57.5258	0.9626	44.1193	0.9221
	0.7	52.2322	0.9575	37.7875	0.9013
	0.8	48.1042	0.9536	32.7296	0.8798
Average (0.1 ~ 0.4)		91.5119		83.4133	

## Data Availability

Data are contained within the article.
